# Radiation synthesis of MXene/Ag nanoparticle hybrids for efficient photothermal conversion of polyurethane films

**DOI:** 10.1039/d3ra02799f

**Published:** 2023-05-18

**Authors:** Chenghao Zhang, Youwei Zhang, Xiaoxia Gu, Cankun Ma, Yicheng Wang, Jing Peng, Maolin Zhai, Minxuan Kuang, Huiling Ma, Xiuqin Zhang

**Affiliations:** a Beijing Key Laboratory of Clothing Materials R & D and Assessment, Beijing Engineering Research Center of Textile Nanofiber, School of Materials Science & Engineering, Beijing Institute of Fashion Technology Beijing 100029 China hlma@bift.edu.cn clyzxq@bift.edu.cn; b Beijing Institute of Aeronautical Materials Beijing 100095 China; c Beijing National Laboratory for Molecular Sciences, Department of Applied Chemistry and the Key Laboratory of Polymer Chemistry and Physics of the Ministry of Education, College of Chemistry and Molecular Engineering, Peking University Beijing 100871 China

## Abstract

Flexible conductive films based on light-to-heat conversion are promising for the next-generation electronic devices. A flexible waterborne polyurethane composite film (PU/MA) with excellent photothermal conversion performance was obtained by combination of PU and silver nanoparticle decorated MXene (MX/Ag). The silver nanoparticles (AgNPs) uniformly decorated on the MXene surface by γ-ray irradiation induced reduction. Because of the synergistic effect of MXene with outstanding light-to-heat conversion efficiency and the AgNPs with plasmonic effect, the surface temperature of the PU/MA-II (0.4%) composite with lower MXene content increased from room temperature to 60.7 °C at 5 min under 85 mW cm^−2^ light irradiation. Besides, the tensile strength of PU/MA-II (0.4%) increased from 20.9 MPa (pure PU) to 27.5 MPa. The flexible PU/MA composite film shows great potential in the field of thermal management of flexible wearable electronic devices.

## Abstract 2

AgNPs decorated MXene hybrid (MX/Ag) with excellent photothermal conversion performance was prepared by γ-ray induced reduction at room temperature.

## Graphical abstract

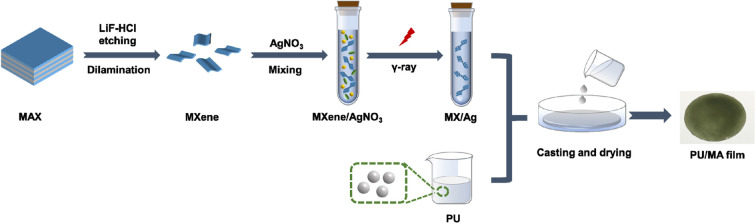

## 1 Introduction

With economic development and population growth, the global energy demand has increased significantly. Finding reliable, low-cost and high-profit renewable energy to meet the future energy needs is important. Solar energy is a promising natural energy source. The Earth receives about 4.4 × 10^14^ MJ h^−1^ of solar energy, which far exceeds the global annual energy consumption.^1,2^ Solar photothermal conversion technology is a simple, direct and effective way to utilize solar energy. It has been gradually applied in steam power generation,^3^ seawater desalination,^4^ and photothermal therapy.^5^ However, exploiting solar energy on a large scale is difficult because of its low energy density and discontinuity. Therefore, the conversion of solar energy to heat has always been the focus of international attention.^6^

The utilization of photothermal conversion materials has been considered as an effective approach to converse solar energy to thermal energy. Polymer-based photothermal conversion materials have the advantages of stable photothermal conversion performance,^7^ high energy storage,^8^ flexible tensile resistance^9^ and so on. They are composed of photothermal fillers and polymer matrix. In general, photothermal fillers have high absorption capacity and photothermal conversion efficiency over the entire solar spectrum. Noble metal nanoparticles (such as gold and silver nanoparticles) are common photothermal conversion material due to their distinctive nanostructures with localized surface plasmon resonance (LSPR) effects.^10–12^ The match of the incident photon frequency and the free electrons of noble metal nanoparticles is beneficial to the adsorption of photon energy and LSPR effect.^13,14^ Chen *et al.* explored the photothermal conversion properties of gold (Au), silver (Ag), and mixed gold-silver (Au–Ag) nanoparticles. Au–Ag mixed nanoparticles (1.75–0.15 ppm) had the highest photothermal conversion efficiency (30.97%).^15^ Chen *et al.* prepared a photothermal conversion material that exhibits high absorption in the visible region by adjusting the molar ratio of Ag to Au precursor.^16^ Ultrathin layered double hydroxide loaded Ag@Ag_2_O core–shell nanoparticles prepared by Li *et al.* exhibited the high photothermal conversion efficiency of 76.9%.^17^ Yuan *et al.* proposed a one-pot method to prepare superparamagnetic iron oxide-enclosed hollow gold nanoshell (SPIO-HGNS) with tunable optical absorption properties.^18^ Although the metal nanoparticles had good photothermal conversion performance, the limitation of their narrow absorption band requires high filler loadings in the polymer matrix, which increases the cost and limits their development.^19^

MXene, a new two-dimensional (2D) transition metal carbides or carbonitrides,^20–22^ is widely used as a photothermal filler in applications of photothermal conversion in smart electronics due to its excellent photothermal properties. Li *et al.* designed a set of aqueous droplet light heating system and mathematical program, and prepared the MXene film by vacuum filtration method. The photothermal conversion efficiency of MXene (Ti_3_C_2_) is 100%, the light-to-water-evaporation efficiency of MXene film reached 84%.^23^ A solar steam generator based on a three-dimensional MXene architecture was prepared by Zhao *et al.*, which showed a solar evaporation efficiency of 88.7%.^24^ A Janus membrane with excellent solar evaporation efficiency (86.4%) was obtained by self-assembly of porphyrin hydrophobic layer on MXene surface.^25^

In addition, MXene is easy to be modified with other materials, such as semiconductors, metal nanoparticles,^26^ metallic nanowires,^27^ carbon nanomaterials,^28^ or polymers, due to its large specific surface area, good hydrophilicity, rich functional groups.^11^ Liu *et al.* prepared AgNW@MXene modified polyester fabric (AM-textile) by a simple alternating impregnation method. The composite fabric rapidly reached 224 °C within 10 s under near-infrared laser (100 mW), showing excellent photothermal response.^29^ Zhou *et al.* developed a flexible and multifunctional transparent conductive film by combining MXene and AgNW with polycarbonate by simple spraying method. When the film was exposed to 1 solar radiation (100 mW cm^−2^) for 150 s, the surface temperature was 40.2 °C, showing an effective photothermal response.^30^ The combination of 2D MXene and zero-dimensional (0D) metal nanoparticles can also improve the efficiency of photothermal conversion. Gold nanorods (GNRs) decorated Ti_3_C_2_ nanosheets (Ti_3_C_2_@GNRs) were obtained by *in situ* growth and self-assembly, and it exhibited a higher photothermal conversion efficiency (45.89%) than that of MXene (31.44%).^31^ The water evaporation efficiency of Au/Ti_3_C_2_/PF membrane reached 83.63%, which were attributed to the increase of the light absorption by Au nanoparticles.^32^ Copper sulfide (CuS) nanoparticles decorated Ti_3_C_2_T_*x*_ nanosheets (Ti_3_C_2_T_*x*_@CuS) were obtained by hydrothermal method, and it exhibited a higher photothermal conversion temperature.^33^ Wang *et al.* prepared fSiO_2_/MXene@Au-WPU composites by a simple spray method, which showed high photothermal conversion rate, superhydrophobicity and acid (alkali) resistance.^34^ Jia *et al.*, prepared a multifunctional flexible film with good photothermal ability by continuously spraying silver particles and MXene suspension on waterborne polyurethane film, supplemented by hot pressing.^35^ Jiao *et al.*, prepared MXene@Ag by direct reduction, and then obtained CNF/MXene@Ag composite membrane by vacuum filtration. The composite film exhibited excellent photothermal properties, and the surface temperature of the film can rise to 48 °C for 210 s under 55 W light irradiation.^36^

Gamma irradiation technology is a facile technique for the modification of nanomaterials. There are some advantages, such as environmentally friendly, the higher product purity, lower energy consumption, and without the need of adding initiators, reducing agents, or other harmful chemicals to the reaction system.^37^ Furthermore, controllable synthesis of nanoparticles can be achieved by adjusting absorbed dose or dose rate during irradiation.^38^ There have been some reports on the synthesis of modified 2D nanomaterial with inorganic nanoparticles by γ-ray irradiation in recent years. MXene/MoSx hybrid materials were synthesized with high hydrogen evolution reaction performance by gamma irradiation.^39^ Hareesh *et al.* prepared reduced graphite oxide nanocomposites loaded with silver nanoparticles (Ag-rGO) by gamma radiation-assisted method.^40^ And graphene oxide-based magnesium oxide nanocomposites were successfully prepared by gamma irradiation.^41^

Herein, silver nanoparticles (AgNPs) decorated MXene (MX/Ag) hybrids was prepared by γ-ray induced reduction at room temperature. The obtained MX/Ag was characterized by XRD, TEM and XPS. Furthermore, a waterborne polyurethane composite film (PU/MA) were prepared and its photothermal conversion properties were also investigated. The prepared PU/MA film is expected to be applied as a smart electronic material.

## 2 Experimental

### 2.1 Materials

Ti_3_AlC_2_ (MAX) was purchased from 11 Technology Co., Ltd. Lithium fluoride (LiF, AR), hydrochloric acid (HCl, AR) and isopropanol (AR) were obtained by Shanghai Macklin Biochemical Co., Ltd. Silver nitrate (AgNO_3_, AR) was obtained by Beijing Tongguang Fine Chemical Co., Ltd. Waterborne polyurethane (PU, Impranil DL1380) was obtained from Shanghai Yuanhe Chemical Co., Ltd.

### 2.2 Synthesis of MXene nanosheets

The MXene nanosheets were synthesized based on a LiF and HCl etching method. First, 4 g of LiF was slowly added to 50 mL of HCl, and then 1.5 g of MAX powder was slowly added to the above solution and stirred at 40 °C for 72 h. After this, the mixture was washed with deionized water until the pH reached about 7. MXene nanosheets were obtained through ultrasonication under the protection of N_2_ and centrifuged at 3500 rpm for 30 min. The concentration of the MXene colloidal dispersion was obtained by weighing the mass of the dry sample for the fixed volume.

### 2.3 Synthesis of MX/Ag hybrids

MX/Ag hybrids were synthesized by γ-ray induced reduction of AgNO_3_. Typically, AgNO_3_ were added to 10 mL deionized water to form 5, 10 and 20 mmol L^−1^ solution. Then, 10 mL of MXene (5 mg mL^−1^) and 2.1 mL of isopropanol were added. The obtained solution was deaerated by N_2_ gas, sealed, and irradiated using a^60^Co source at the College of Chemistry and Molecular Engineering of Peking University. The dose was 50 kGy and the dose rate was 200 Gy min^−1^. Then the product was separated by filtration, washed with deionized water several times, and the concentration of the dispersion was measured. According to the different concentrations of AgNO_3_ solution (2.5, 5, and 10 mmol L^−1^) in the reaction system, the products were named as MA-I, MA-II, and MA-III, respectively.

### 2.4 Preparation of PU/MX and PU/MA nanocomposite films

**Scheme 1:**
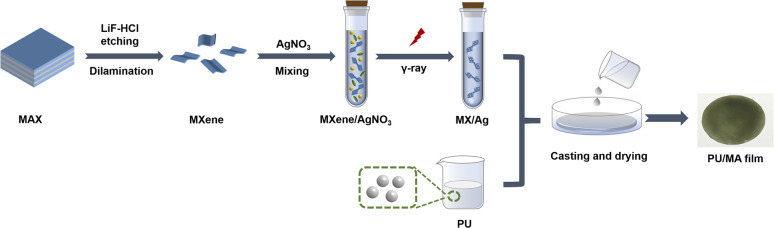
Schematic diagram of the synthetic route of PU/MA film.

MX/Ag and MXene aqueous solution were mixed with a PU solution (solid content of 60 wt%). After ultrasonication for 15 min (270 W), the mixtures were coated on the glass substrate and dried at 60 °C for 2 h to prepare the composite films. The MX/Ag hybrids was 0.1, 0.2, 0.3 and 0.4 wt% of PU resin, and the obtained composite films were recorded in Table 1. And the fabrication process is demonstrated in (Scheme 1).

**Table 1:** Preparation conditions and the weight percent of fillers

Name	Filler	Weight percent (wt%)
PU/MX (0.1%)	MXene	0.1
PU/MA-I (0.1%)	MA-I	0.1
PU/MA-II (0.1%)	MA-II	0.1
PU/MA-III (0.1%)	MA-III	0.1
PU/MA-II (0.2%)	MA-II	0.2
PU/MA-II (0.3%)	MA-II	0.3
PU/MA-II (0.4%)	MA-II	0.4

### 2.5 Characterization

The samples were characterized by X-ray diffraction (XRD, Rigaku D/Max 2400 diffractometer, Japan) with Cu Kα source. The structure and morphology of the products were characterized by scanning electron microscopy (SEM, JEOL JSM-7500F, Japan) and transmission electron microscopy (TEM, JEM-2100F, Japan). X-ray photoelectron spectroscopy (XPS, Kratos Analytical, UK) was measured on an Axis Ultra X-ray photoelectron spectrometer using monochromatic Al Kα radiation. The UV-VIS-NIR absorbance spectra were collected using a Shimadzu UV-2600 absorption spectrophotometer. Xenon light intensity was tested by a solar power meter (SM206-SOLAR, China). The surface temperature of the composite film is measured by an infrared thermal imager (Testo 869, Germany). The mechanical properties of the composite films were tested at room temperature using a tensile testing machine (Instron-3365, China) at a speed of 100 mm min^−1^. The photo-to-thermal conversion tests was carried out using a xenon lamp in an indoor environment. The PU/MA composite film was placed on a polystyrene foam board. The infrared thermal imager is vertically fixed on the upper left side of the sample, and the vertical distance to the sample surface is 20 cm. A solar power meter and a thermos-hygrometer were used to monitor the solar irradiance, the ambient temperature and relative humidity.

## 3 Results and discussion

**Fig. 1:**
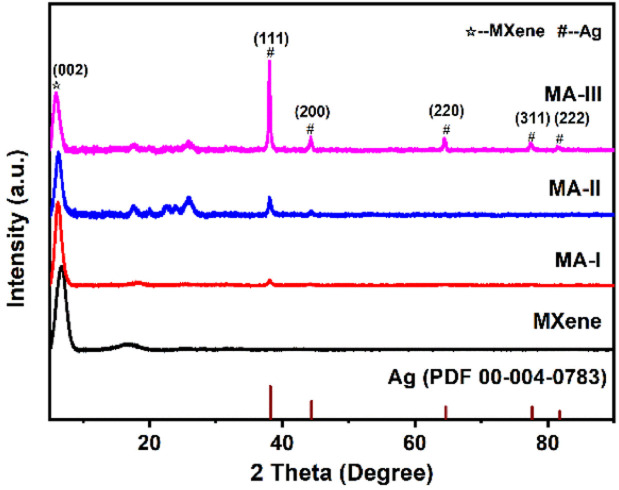
XRD patterns of MXene and MA hybrid materials.

The crystal structure of MXene and MA hybrid materials was confirmed by XRD patterns (Fig. 1). The peak at ∼39° belonging to MAX disappeared, and a new characteristic peak at 6.8° can be indexed to (002) plane of MXene, indicating that MAX was completely etched. After MXene nanosheets were modified by AgNPs, five characteristic diffraction peaks at 38.1°, 44.3°, 64.5°, 77.4° and 81.6° are ascribed to the (111), (200), (220), (311), (222) crystal planes of AgNPs, respectively. The peak intensity increases with the increased AgNPs content, while the (002) peak intensity of MXene decreases because of the introduction of AgNPs. In addition, compared with MXene, the (002) characteristic peak of MA-III hybrid material shifts to 5.9°, which is lower than that of MXene. This is due to the AgNPs decorated between the MXene layers increasing the interlayer spacing. And the AgNPs between the layers can prevent the restacking of MXene nanosheets.

**Fig. 2:**
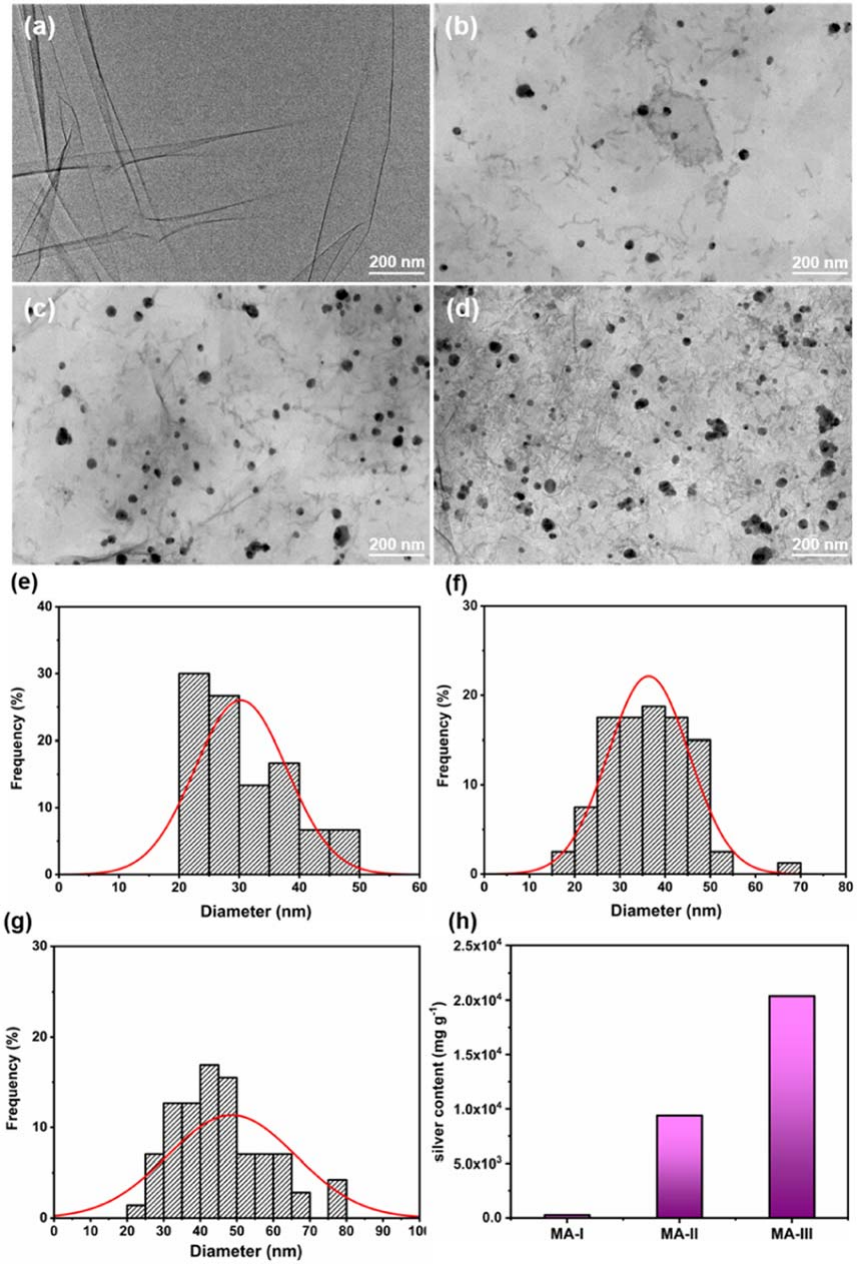
TEM images and the size distribution of AgNPs for (a) MXene, (b and e) MA-I, (c and f) MA-II and (d and g) MA-III. (h) The silver contents of MA-I, MA-II and MA-III.

Fig. 2 shows the TEM images of MXene, MA-I, MA-II and MA-III hybrid materials. The surface morphology of MXene nanosheet and the AgNPs on the MXene surface can be observed. A single MXene sheet with wrinkle structure is shown in Fig. 2a, which is an intrinsic feature of MXene. Fig. 2b–g is the TEM images and the size distributions of AgNPs for MA-I, MA-II and MA-III samples. The spherical AgNPs (diameter, 20–80 nm) are more uniformly distributed on MXene surface. For MA-I, a small amount of AgNPs loaded on the surface of MXene, with an average diameter of 30.3 nm (Fig. 2e). In addition, with the increase of AgNO_3_ concentration, the average diameter of AgNPs for MA-II and MA-III slightly increased to 36.4 and 48.6 nm, and a certain degree of agglomeration occurred. The silver contents of MA-I, MA-II, and MA-III samples were measured by ICP (Fig. 2h). When the AgNO_3_ concentration in the reaction system were 2.5, 5, and 10 mmol L^−1^, the corresponding silver contents of MA-I, MA-II, and MA-III were 2.8 × 10^2^, 9.4 × 10^3^ and 2.0 × 10^4^ mg g^−1^, respectively. The silver content increased with the increase of AgNO_3_ concentration.

**Fig. 3:**
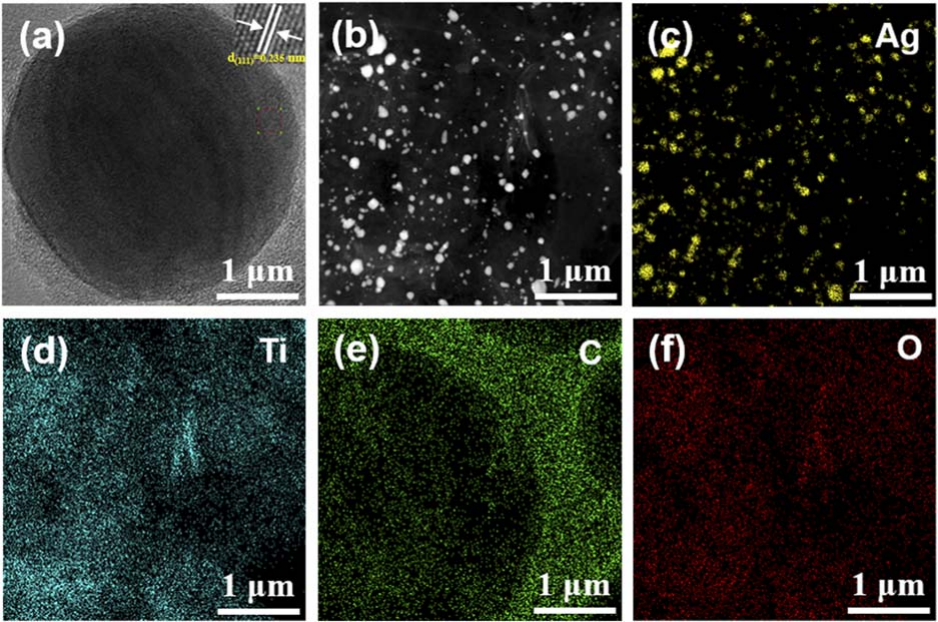
(a) High-resolution TEM images of the MA-II. (b–f) EDS elemental mapping images of Ag, Ti, C and O for the MA-II.

The MA-II samples were further tested by high-resolution TEM and EDS (Fig. 3). The interplanar spacing of the nanoparticle is 0.235 nm, which is ascribed to the (111) plane of Ag. In addition, Ag, Ti, C and O were uniformly distributed in the sample (Fig. 3c–f), which further verified the uniform dispersion of AgNPs. Therefore, MXene decorated with AgNPs hybrids can be simply obtained by γ-ray irradiation.

**Fig. 4:**
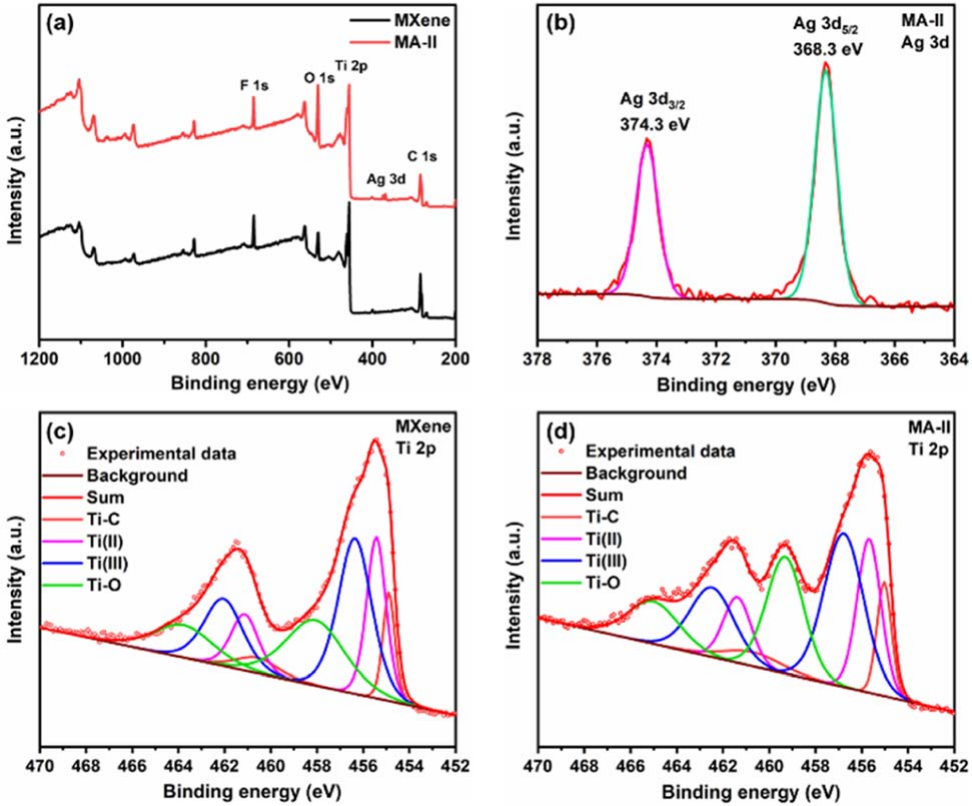
(a) Low-resolution XPS spectra of MXene and MA-II, (b) Ag 3d XPS spectra of MA-II, Ti 2p XPS spectra of (c) MXene and (d) MA-II.

The different chemical environments of MXene and MA-II nanosheets are investigated by XPS spectra (Fig. 4). The low-resolution XPS spectra of MXene and MA-II hybrid exhibit the peaks of C, O, Ti and F. Compared with MXene spectrum, a new peak corresponding to Ag 3d at 368 eV appears, demonstrating the existence of AgNPs in the hybrid material. The Ag 3d spectrum of MA-II hybrid material shows two peaks at 368.3 eV and 374.3 eV, belonging to Ag 3d_5/2_ and Ag 3d_3/2_, respectively (Fig. 4b). Furthermore, the spin energy separation between the two peak is about 6.0 eV. It is indicated that AgNPs were loaded on MXene nanosheets.^42^ Ti 2p XPS spectra of MXene and MA-II exhibit four characteristic peaks located at 455.2, 455.6, 456.8 and 459.3 eV, which are ascribed to Ti–C, Ti(ii), Ti(iii) and Ti–O bonds, respectively (Fig. 4c and d). Compared with MXene, the Ti–O peak intensity of MA-II hybrid increases significantly, indicating the existence of oxidation in MA-II under γ-ray irradiation. This may be due to the fact that Ti atoms on the surface of MA-II are oxidized by some oxidizing free radicals produced by H_2_O radiolysis.

**Fig. 5:**
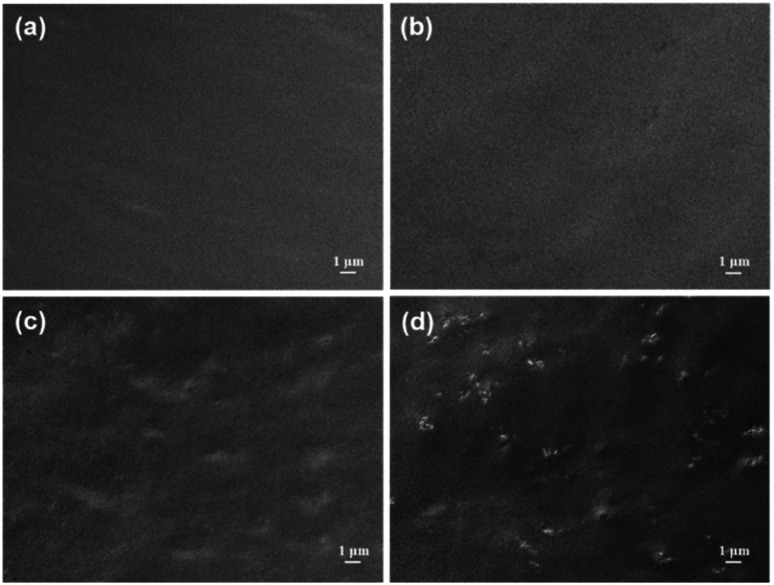
SEM images of (a) PU, (b) PU/MX (0.1%), (c) PU/MA-II (0.1%) and (d) PU/MA-II (0.4%).

The dispersion of MA in PU/MA composites was observed by SEM in Fig. 5. The brittle fracture section of the pure PU composite film is smooth and flat. For PU/MX (0.1%), there was no obvious change in the cross section of the polymer. When 0.1 wt% MA-II was added to PU, the roughness of the PU/MA-II (0.1%) surface slightly increased. When the addition of MA-II increased to 0.4 wt% (PU/MA-II (0.4%)), some bulges were observed on the cross section of the samples, implying that the roughness of PU composites surface increased. Besides, MA-II is well dispersed in PU, which may be attributed to the interaction between PU and the polar groups (–OH) on MXene surface.

**Fig. 6:**
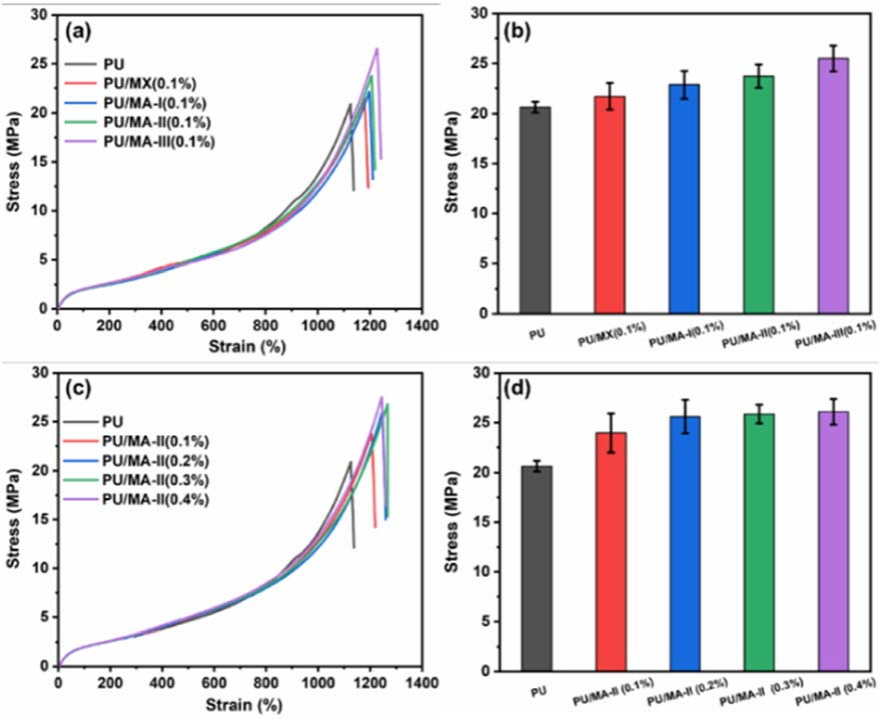
(a) Stress–strain curves of PU, PU/MX (0.1%), PU/MA-I (0.1%), PU/MA-II (0.1%) and PU/MA-III (0.1%) composites, (b) stress–strain curves of PU, PU/MA-II (0.1%), PU/MA-II (0.2%), PU/MA-II (0.3%) and PU/MA-II (0.4%), (c) stress strength of PU/MX and PU/MA composites.

Mechanical property plays a key role in evaluating the potential applications of polymer nanocomposites. The stress–strain curves of PU nanocomposites are exhibited in Fig. 6. Compared with pure PU, the tensile strength of PU/MA-III (0.1%) samples increased by 27.0% from 20.9 MPa to 26.6 MPa. It is attributed to the addition of AgNPs, which attached on MXene surface impeding the stacking of MXene sheets and promoting their dispersion in the PU matrix.^39,43^ The tensile–strain curve of PU composites with different MA-II content are shown in Fig. 6c and d. The tensile strength of the PU composites slightly increased with the increasing MA-II content. The tensile strength of PU/MA-II (0.4%) increased by 31.6%, from 20.9 MPa of pure PU to 27.5 MPa. The increased tensile strength of PU/MA composites prepared in this paper is much higher than other work.^44,45^ The improved mechanical properties of PU/MA composites are mainly attributed to the abundant interfacial interactions between the inorganic nanomaterial and polymer networks.^46^ MXene and MA-II hybrid materials not only absorb the stress, but also transfer the loadings to the adjacent polymer chains and nanofillers due to the good compatibility between PU matrix and MA-II hybrid. Thereby the tensile strength of the composite improved.^47^ In addition, the elongation at break of PU nanocomposites slightly increased with the increasing of MA-II content. The elongation at break of PU/MA-II (0.1%) nanocomposites increased by 7.2%, from 1125% of PU to 1206%. When the content of MA-II increased to 0.4%, the value reached 1245%, which may be due to the wrinkled morphology of PU/MA-II in the PU nanocomposites.

**Fig. 7:**
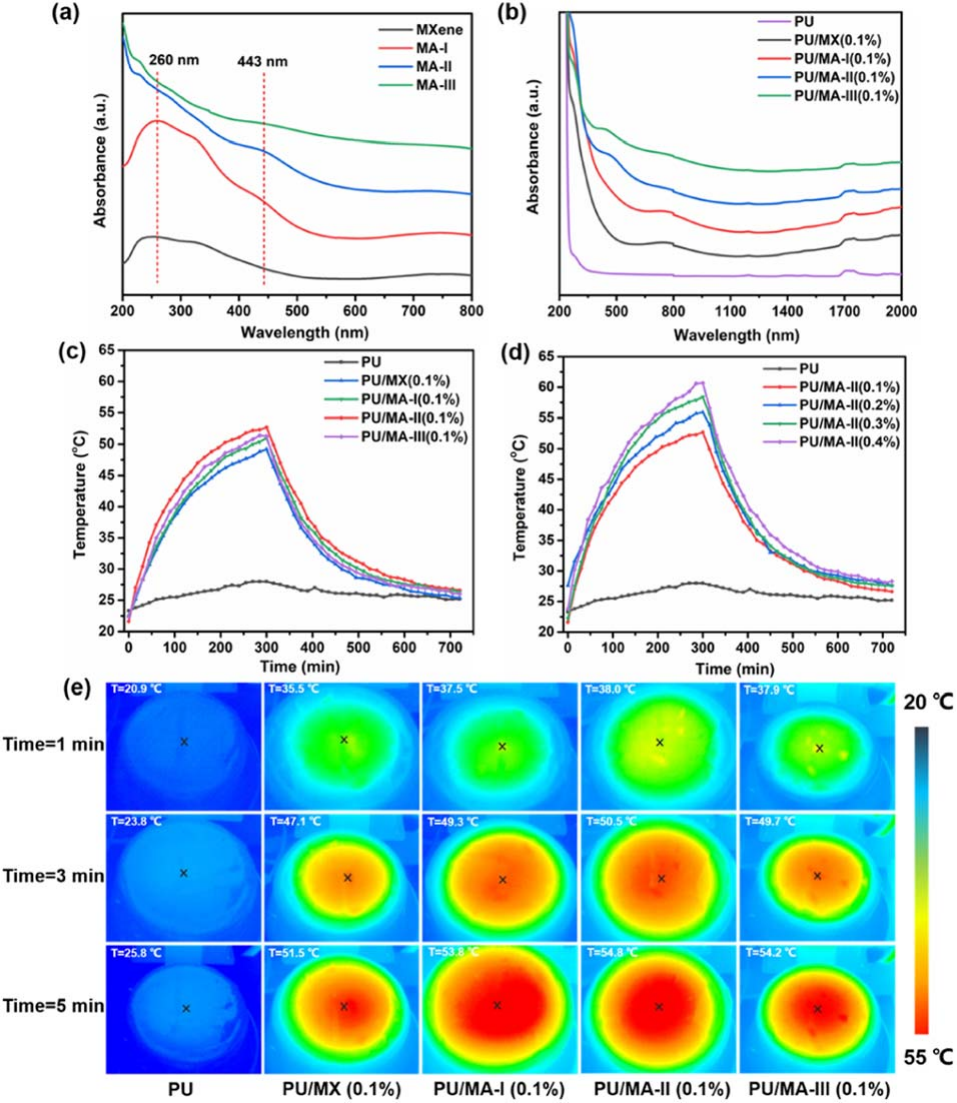
(a) UV-vis spectra of MXene, MA-I, MA-II and MA-III aqueous solutions. (b) UV-VIS-NIR spectra and (c) the surface temperature evolution of PU, PU/MX (0.1%), PU/MA-I (0.1%), PU/MA-II (0.1%), and PU/MA-III (0.1%). (d) PU and PU/MA-II composites with 0.1, 0.2, 0.3, and 0.4 wt% MA-II. (e) Infrared thermal images of PU, PU/MX, and PU/MA composites under 85 mW cm^−2^ irradiation.

The UV-vis spectra of MXene and MA-I, MA-II and MA-III nanohybrids are shown in Fig. 7a. MXene exhibits a unique absorption peak at approximately 260 nm and 770 nm. It can be assigned to the band gap energy of MXene. In addition, MA-I, MA-II and MA-III hybrid materials showed a new peak at ∼443 nm in the visible region. And the peak intensity increased with the increasing AgNPs content, which is ascribed to the surface plasmon resonance effect of AgNPs loaded on MXene surface.^48^ The UV-VIS-NIR spectra of PU, PU/MX (0.1%), PU/MA-I (0.1%), PU/MA-II (0.1%), and PU/MA-III (0.1%) at 200–2000 nm were further studied (Fig. 7b). Compared with pure PU film, the other four samples also showed the characteristic absorption peaks of 260 nm and 770 nm, and exhibit good absorption in NIR range (wavelength: 1100–2000 nm). In addition, with the increase of AgNPs content, PU/MA-II (0.1%) and PU/MA-III (0.1%) showed the characteristic absorption peaks of AgNPs at 440 nm. The increased absorption intensity of the hybrid material is beneficial to the improvement of the photothermal properties of PU composites.

The photothermal conversion abilities of PU nanocomposite films were confirmed by recording the surface temperature (tested by infrared thermal imager) of PU composite exposed to xenon lamp (simulated solar light source with light intensity of 85 mW cm^−2^). As shown in Fig. 7c, PU film exhibits a negligible photothermal conversion performance with a slight rise of ∼4.7 °C from 23.3 °C to 28.0 °C when the film was irradiated for 5 min. By contrast, with the same irradiated time, the surface temperatures of PU/MX (0.1%), PU/MA-I (0.1%), PU/MA-II (0.1%) and PU/MA-III (0.1%) composite films significantly increased to 49.2, 51.0, 52.7 and 51.3 °C, respectively. This is ascribed to the effective photothermal conversion of PU composite with the addition of MA hybrid material. Besides, PU/MA-II composite film exhibits the highest temperature among the other composite films under the same irradiated time, which is attributed to the synergistic interaction between AgNPs and MXene under this condition. Meanwhile, the surface temperature of PU/MA films with different MA-II contents were provided in Fig. 7d. The surface temperature of PU/MA-II (0.1%), PU/MA-II (0.2%), PU/MA-II (0.3%) and PU/MA-II (0.4%) increase obviously from room temperature to 52.7, 55.9, 58.4 and 60.7 °C, respectively. It is worth noting that PU/MA-II (0.4%) exhibited the most excellent photothermal conversion performance. Under the same irradiated conditions, the surface temperature was 36.9 °C higher than that of the PU film. And the infrared thermal imager was applied to visually record the surface temperature of these composites films with different photothermal fillers (Fig. 7e). The photothermal conversion performances of other typical MXene-based or PU nanocomposites film are summarized in Table 2. PU/MA-II (0.4%) exhibits relatively excellent photothermal conversion properties under lower energy density.

**Table 2:** Comparison of the increased temperature after the irradiation with other photothermal conversion materials

Photothermal materials	Light intensity (mW cm^−2^)	The temperature increased (°C)	Ref.
PDA-WPU	100	30.3	49
WPU/NR/MXene	100	∼42	50
WPU@MXene/PEG	100	∼25	9
Au/Ti_3_C_2_/PF	200	∼23.1	32
Ag/MXene films	100	∼38.2	35
PU/MA-II (0.4%)	85	36.9	This work

The temperature of PU/MA composite film with lower MA content rapidly increases under the light irradiation, which is due to the synergistic photothermal conversion of MXene nanosheets^23^ and the AgNPs.^51^ Firstly, the light can be absorbed by PU/MA composites to convert heat due to the highly efficient photothermal conversion efficiency of MXene.^23,52^ Secondly, the dipole resonance coupling between AgNPs and simulated sunlight promotes the absorption of incident light in a wider wavelength range, and converts it into heat, which is due to the plasmon resonance effect of AgNPs.^15,53^ Finally, the photothermal filler MA can be well dispersed in PU matrix, which is beneficial to the effective heat conversion from 2D MXene and 0D AgNPs to PU polymer matrix, thereby increasing the temperature of nanocomposite film. And the well dispersion of nano fillers in polymer matrix is ascribed to the good hydrophilicity of MXene nanosheets.^54^

**Fig. 8:**
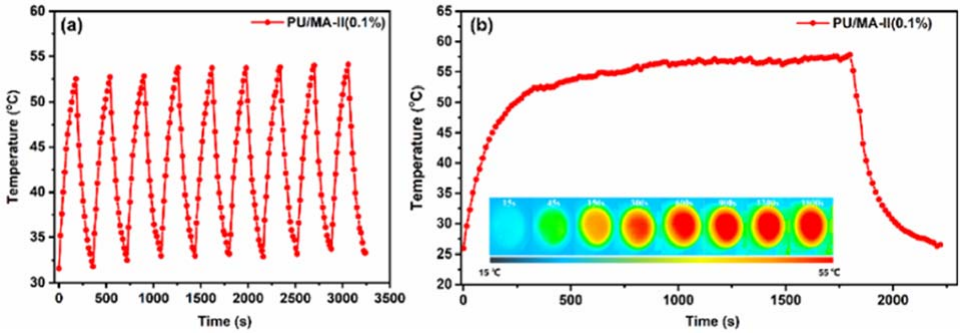
(a) Repeated on-off light cycles, (b) long-term irradiation for 0.5 h with corresponding IR images (insets) of PU/MA-II (0.1%) under 85 mW cm^−2^ irradiation.

The heating stability is also an important property for photothermal materials. Fig. 8a shows the repeated on-off light cycles of PU/MA-II (0.1%) under 85 mW cm^−2^ irradiation. The cycle curve of PU/MA-II (0.1%) exhibits the stable and regular temperature change corresponding to the on-off light cycles, demonstrating the distinguished heating cycling stability of the PU composites film. Besides, the long-term (0.5 h) heating stability of PU/MA-II (0.1%) under the same light irradiation is shown in Fig. 8b. When the light irradiation time is extended to 0.5 h, the surface temperature of PU/MA-II (0.1%) remains basically constant, which is attributed to the high chemical stability of MA-II and the well dispersion of MA-II in the PU matrix.

## 4 Conclusions

MX/Ag hybrid materials were prepared by a simple and environmentally friendly approach by γ-ray induced reduction at room temperature. The AgNPs was uniformly decorated on MXene surface and enlarged the interlayer spacing, which is beneficial for the preventing of the aggregation of MXene. Furthermore, MX/Ag hybrid materials was efficient in improving the photothermal conversion and mechanical properties of PU. The tensile stress of PU/MA-II (0.4%) reached 27.5 MPa with a lower filler content. And the surface temperature of PU/MA-II (0.4%) composite increased to 60.7 °C at 5 min under 85 mW cm^−2^. The improved properties of the nanocomposites are ascribed to the synergistic light-to-heat conversion effect of MXene and AgNPs, and the well dispersion of MX/Ag in PU matrix. The obtained material has excellent mechanical properties, photothermal conversion performance and good cycle stability, which is expected to be used in the flexible wearable electronic devices.

## Author contributions

H. Ma, X. Zhang and M. Zhai guided the project. C. Zhang and Y. Zhang were responsible for the design of experiments. C. Zhang and X. Gu performed PU/MX and PU/MA composite films fabrication. C. Ma, Y. Wang and M. Kuang carried out the structural characterization and analysed the data. C. Zhang and J. Peng conducted the photothermal conversion performance measurements. H. Ma and C. Zhang wrote the initial draft of the manuscript, and the manuscript was revised by M. Cui. All authors have given approval to the final version of this manuscript.

## Conflicts of interest

There are no conflicts to declare.

## Supplementary Material
